# Relaxed Stiffness of Lower Extremity Muscles and Step Width Variability as Key Differences Between Sarcopenia and Dynapenia in Community-Dwelling Older Adults: A Cross-Sectional Study

**DOI:** 10.3390/life16010042

**Published:** 2025-12-26

**Authors:** Jiseul Park, Youngsook Bae

**Affiliations:** 1 Department of Health Science, Major in Physical Therapy, Graduate School, Gachon University, Incheon 21936, Republic of Korea; 2 Department of Physical Therapy, College of Medical Science, Gachon University, 191 Hambangmoe-ro, Yeonsu-gu, Incheon 21936, Republic of Korea

**Keywords:** dynapenia, gait variability, muscle stiffness, older adults, sarcopenia, shear-wave elastography

## Abstract

*Background and Objectives*: Sarcopenia and muscle wasting contribute significantly to functional decline in older adults, but differences in lower extremity muscle stiffness and gait variability between these groups are not yet fully understood. This study aimed to compare gait variability, and lower extremity muscle stiffness during contraction and relaxation in community-dwelling older adults classified as non-diseased, sarcopenic, and dynapenic. *Materials and Methods*: This cross-sectional study included 164 community-dwelling older adults classified as non-diseased, dynapenic, or sarcopenic, based on handgrip strength, 5-time sit-to-stand test, and skeletal muscle index. Spatiotemporal gait variability was measured at the participants’ preferred speed. Moreover, muscle thickness, as well as the contractile and relaxed stiffness, were measured for the rectus femoris (RF), biceps femoris (BF), tibialis anterior (TA), gastrocnemius medialis (GAmed), and lateralis (GAlat). *Results*: In dynapenic and sarcopenic groups, gait variability increased across most parameters, but only the step width coefficient of variation differed significantly between the dynapenic and sarcopenic groups. Contractile stiffness of the RF, BF, and GAlat was lower in both groups, with additional GAmed stiffness reduction in the sarcopenic group. Relaxed stiffness of the BF and GAmed was significantly higher in the sarcopenic group than in the dynapenic group. *Conclusions*: This study identified differences in muscle thickness, stiffness, and gait variability among non-diseased, dynapenic, and sarcopenic older adults. Step width variability, GAmed contractile stiffness, and BF and GAmed relaxed stiffness emerged as potential early indicators for distinguishing dynapenia from sarcopenia. These findings highlight the importance of assessing muscle quality—including both mass and stiffness characteristics—to better characterize early stages of age-related muscle decline and to inform targeted intervention strategies.

## 1. Introduction

Sarcopenia in older adults is characterized by an increased risk of falls and diminishing quality of life [1]. The criteria for diagnosing sarcopenia in Asian populations were established by the Asian Working Group for Sarcopenia [2] and included assessments of skeletal muscle mass and physical function with thresholds for hand grip strength (HGS) and the five-time sit-to-stand test (5TSTS). These diagnostic standards are widely applied across Asia, including in Korea. In contrast, dynapenia, characterized by a decline in muscle strength due to aging and defined as the loss of muscle strength [3], somewhat differs from sarcopenia, which is a decline in muscle strength and decreased muscle mass or lean body mass [4].

Sarcopenia and dynapenia are characterized by decreased muscle strength. For older adults, maintaining and/or improving muscle strength is important for maintaining physical function [5]. Furthermore, by maintaining muscle strength, functional limitations and mortality may reduce, regardless of muscle mass [6]. Thus, muscle strength is not solely dependent on muscle mass, and as we age, strength declines at a faster rate than muscle mass, suggesting a decline in muscle quality [7,8] and that declining muscle mass and strength need to be examined separately. Therefore, to assess physical function in older adults, it is more important to evaluate dynapenia rather than sarcopenia.

Indeed, limitations of physical function and loss of physical independence due to falls negatively impact the quality of life [9], while the loss of muscle strength reflects a decline in muscle quality [7]. Specifically, deterioration in the lower-limb muscle quality has been associated with impairments in mobility and other physical functions [10]; also, physical function correlates with gait variability (GV) [11]. Because GV is closely related to gait stability in older adults, it may also correlate with muscle function, including muscle strength [12]. Given the strong association between gait variability and functional decline, identifying gait parameters related to variability may provide clinically meaningful indicators for the early detection of mobility impairments in older adults.

Dynapenia involves changes in the contractile properties or neural function of muscles, highlighting the necessity of muscle quality monitoring, including decreased contractility [13]. Thus, lower-extremity muscle strength and quality may be accurate indicators of functional decline. Moreover, distinguishing between sarcopenia and dynapenia requires a deeper understanding of muscle quality rather than the mere comparison using muscle mass. Therefore, to assess sarcopenia and dynapenia, preferably, muscle function, including muscle strength, quality, and GV, should be evaluated.

Recently, skeletal muscle ultrasonography has gained recognition as a reliable and validated tool for assessing muscle mass in the diagnosis of sarcopenia [14,15]. In addition to traditional measures of muscle thickness, shear wave elastography (SWE) has emerged as a promising technique for quantitatively evaluating passive and active muscle properties [16]. Aging is associated with a decrease in skeletal muscle stiffness as measured using SWE, which correlates significantly with strength loss [17]. Janczyk et al. [18] assessed the stiffness in the gastrocnemius and rectus femoris (RF) muscles to evaluate sarcopenia-related changes in the lower limbs. Reduced gastrocnemius muscle thickness has been associated with decreased muscle mass [19], while contractile stiffness of the gastrocnemius medialis (GAmed) has been linked to impaired balance ability [20]. Furthermore, RF thickness is recommended as an indicator of sarcopenia assessment [21]. These findings suggest muscle stiffness as an objective biological marker to evaluate functional muscle changes in older adults. However, studies directly comparing muscle function between sarcopenia and dynapenia are limited.

Despite the clinical importance of early differentiation between sarcopenia and muscle weakness, little is known about whether biomechanical parameters, such as gait variability or muscle stiffness, can serve as practical indicators of early intervention or fall prevention strategies. Therefore, identifying phenotype-specific muscle and gait characteristics could support targeted rehabilitation approaches and improve clinical decision-making in older adults at risk for motor decline.

Therefore, the primary objective of this study was to measure muscle thickness and stiffness, as well as spatiotemporal gait parameter variability during contraction and relaxation of the RF, biceps femoris (BF), tibialis anterior (TA), GAmed, and lateralis (GAlat) muscles in older adults aged 65 years or older. The goal of this study was to investigate differences in muscle characteristics and GV among older adults who were either non-diseased or had dynapenic and sarcopenic gaits.

## 2. Materials and Methods

### 2.1. Design and Ethical Considerations

In this cross-sectional observational study, 164 older adults were recruited between September 2023 and January 2024. Before commencing the experimental process, detailed information about the study procedure and safety was provided to the participants who subsequently signed a written informed consent form. This study was approved by the Institutional Review Board of Gachon University (1044396-202308-HR-165-01, approval date: 20 September 2023) and conducted in accordance with the Declaration of Helsinki (revised in 2013). In addition, the reporting of this cross-sectional study adhered to the STROBE (Strengthening the Reporting of Observational Studies in Epidemiology) guidelines.

### 2.2. Participants and Recruitment Process

The participants were recruited by posters pasted in community centers and via telephonic interviews according to the following criteria: (1) community dwelling older adults aged ≥65 (65–90) years who were able to perform activities of daily living with or without assistive devices; (2) not currently experiencing orthopedic problems in the lower extremities or neurological disorders, such as cerebral infarction; and (3) had not undergone surgery within the past 6 months for musculoskeletal diseases of the lower limb or taken any muscle-related medications. Additionally, A score of 23 or less is the generally accepted cutoff indicating cognitive impairment [22], participants with cognitive impairment—defined as a score below 23 on the Korean version of the Mini-Mental State Examination (K-MMSE), or those who were unable to understand or follow the researcher’s instructions were excluded Of the 284 initial older adults recruited, 23 older adults who did not meet the inclusion criteria; 24 were unable to perform the measurement procedures; and 73 met only 5TSTS or HGS criterion, respectively, were excluded.

The sample size, calculated using G*Power version 3.1.9.7 (Heinrich Heine University Düsseldorf, Germany), based on power = 0.8, *α* = 0.05, and effect size = 0.5 on a two-tailed test [23], was 128. To account for potential dropouts and to increase the statistical robustness of the analysis, a total of 164 older adults were ultimately included. The researchers were not blinded to the aim of the study, whereas the outcome assessors were.

### 2.3. Data Collection and Measurements

All data were collected from older adults from a community center and were asked to complete a questionnaire about their health status, following which the muscle mass was measured. Subsequently, thickness, relaxation, and contraction stiffness of the muscles were measured using ultrasound. Finally, the gait variables were measured.

#### 2.3.1. Skeletal Muscle Index (SMI) and Physical Performance

The SMI was measured using a body composition analyzer (InBody 370s, Inbody Inc., Seoul, Republic of Korea) that utilizes bioelectrical impedance analysis and was calculated by dividing the appendicular skeletal muscle mass (kg) by the square of the height (m^2^). Sarcopenia was defined as SMI of <7.0 kg/m^2^ for men and <5.7 kg/m^2^ for women [2].

Physical performance was measured using the HGS and 5TSTS according to the Asian Working Group for Sarcopenia diagnostic criteria [2]. HGS was measured using a hand dynamometer (Jamar Plus+; Sammons Preston, Rolyon, IL, USA). The researcher clarified the measurement procedure to each participant before the assessment. The measurement was taken while the participants sat in a chair, holding the device with their dominant hand, supporting their arm on a table or another stable surface, maintaining a neutral wrist position, and flexing their elbow at 90°. The participant gripped the device as strongly as possible for 3 s, and the average value of three trials was used. This procedure has been reported to have high test–retest reliability [24]. For the 5TSTS, the participants sat on an armless chair with their arms crossed in front of their trunk and their knees bent at 90°. The participants were instructed to stand up from the chair and sit down again as quickly as possible, repeating this motion five times. Each participant performed two trials, and the average of the two times taken to complete both tests was recorded [25].

#### 2.3.2. Gait Parameters and Variability

In this study, the participants were provided with a 2 min adaptation period on the treadmill before data collection to ensure familiarization with the walking environment and promote natural gait patterns. Evidence on most gait parameters during treadmill walking indicates high reliability [26]. Therefore, gait parameters and variability were assessed using the Zebris FDM treadmill system (Zebris Medical GmbH, Max–Eyth–Weg 43, D–88316, Isny, Germany). This system is equipped with an integrated pressure-sensing platform, featuring an effective measurement area of 108.4 × 47.4 cm and containing 7168 sensors, each measuring approximately 0.85 × 0.85 cm. The treadmill walking surface, measuring 150 × 50 cm, was maintained in a horizontal position (0% incline) throughout the measurement sessions.

GV, a sensitive tool, can detect changes in motor control associated with aging and pathological conditions and provides an objective assessment of mobility and functional status [27]. GV was evaluated using the coefficient of variation (CV), a key indicator for quantitatively assessing gait stability and quality. Given the strong correlation between the gait parameter standard deviation (SD) and CV, variability was reported using the SDs. For a reliable assessment of GV, a minimum of 50 steps or at least 1 min of walking data are required [28]. The CV, expressed as a percentage, was calculated as follows: (CV = SD/Mean × 100), and this study included the following primary gait parameters: stride length, step width, velocity, and cadence. Stride length was defined as the distance between consecutive heel contacts, for the same foot, measured in centimeters. Step width was the lateral distance between the midpoints of both feet during walking and was also measured in centimeters. Velocity represents the walking speed, expressed in kilometers per hour, whereas cadence, which is the number of steps taken per minute, reflects the frequency of walking.

#### 2.3.3. Thickness and Stiffness of Muscle

The dominant leg generally exhibits habitual lower extremity function and has been shown to have greater muscle thickness, volume, and better postural control compared to the non-dominant leg, and previous study have measured muscle properties in the dominant leg [29,30,31]. Therefore, in this study, the RF, BF, TA, GAmed, and GAlat muscles of the dominant leg were measured. Assessment was conducted using an RS85 ultrasound device (Samsung Medison, Seoul, Republic of Korea) equipped with a 5–10 MHz linear probe. Muscle thickness, the distance between the superficial and deep fascia, was measured in the transverse plane using B-mode ultrasound. The ultrasound probe was rotated along the muscle fiber direction to evaluate muscle stiffness both at rest and during contraction, using the SWE mode.

For the RF stiffness measurement, the probe was placed precisely at 3/5th point between the anterior superior iliac spine and the superior border of the patella. Resting stiffness was measured in the supine position with the leg fully extended, whereas contracted stiffness was measured in a seated position with the knee flexed at 90° and maximum resistance applied to the ankle [32]. BF stiffness was assessed in the prone position with the probe positioned at the midpoint of the upper 30% of the total thigh length, measured from the ischial tuberosity to the popliteal fossa [33]. Resting stiffness was recorded in this position, while muscle contraction stiffness was measured with the knee flexed at 90°, tibia externally rotated (toes pointing outward), and resistance applied to the lateral side of the ankle [34]. The TA stiffness measurements were conducted in the supine position and evaluated at the one-third point of the distance between the inferior border of the patella and the base of the first metatarsal [35]. Contracted stiffness was measured, while resistance was applied to the proximal phalanx or metatarsal head. GAmed and GAlat stiffnesses were measured at the one-third point of the distance from the popliteus medius to the heel [35]. After measuring the resting stiffness, the contracted stiffness was recorded, while resistance was applied to the middle plantar area.

For the muscle stiffness measurements, the region of interest was defined as a circular area with a 10 mm diameter, which aligned parallel to the muscle fibers. Four regions of interest were placed on each SWE image and the average stiffness value was calculated. All values were recorded in kilopascals. Initially, muscle thickness was measured in a relaxed state, followed by assessment of resting stiffness. The participants were then instructed to contract their muscles for 10 s, during this time, the contracted stiffness was measured. Muscle thickness and stiffness were measured at the same anatomical location. Ultrasound imaging and SWE assessments were conducted by one of two radiologists, each with >5 years of experience in musculoskeletal radiology.

### 2.4. Classifying the Participants into Non-Diseased, Dynapenic, and Sarcopenic Groups

**Figure 1:**
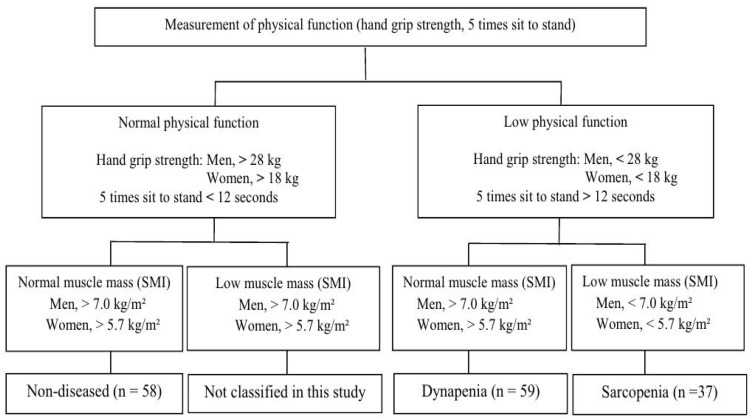
Classified in to non-diseased, dynapenia, and sarcopenia.

Participants were classified into three groups: non-diseased, dynapenic, and sarcopenic, based on grip strength, 5TSTS, and SMI measurements, as previously reported [2,36]. Muscle functional decline was defined as a grip strength of <28 kg for men and 18 kg for women and 5TSTS performance time of ≥12 s. Muscle mass reduction was defined as an SMI of <7.0 kg/m^2^ for men and 5.7 kg/m^2^ for women [2]. Accordingly, the non-diseased group consisted of participants with normal muscle mass and function; dynapenic group included those with normal muscle mass but reduced muscle function; and the sarcopenic group consisted of participants with both reduced muscle mass and function (Figure 1).

Flowchart illustrating the classification of physical function and muscle mass used in this study. Normal physical function with low muscle mass was not included in the analysis; therefore, only three categories were classified: non-diseased (normal physical function with normal muscle mass), dynapenia (low physical function with normal muscle mass), and sarcopenia (low physical function with low muscle mass).

### 2.5. Statistical Analysis

SPSS Statistic for Windows, version 26.0 (IBM Corp., Armonk, NY, USA) was used for the statistical analyses. Descriptive statistics (frequencies, means, and SDs) were used to summarize the participants’ demographic characteristics and baseline measurements, and the normality of continuous variables was assessed using the Shapiro–Wilk test. To compare differences in muscle thickness and stiffness among the non-diseased, dynapenic, and sarcopenic groups, Multivariate Analysis of Covariance (MANCOVA) was performed with sex and age as a covariate to adjust for its potential influence on muscle properties. When significant differences were found, post hoc comparisons were performed using the Bonferroni test. Additionally, a binary regression analysis was conducted to determine whether muscle thickness and stiffness variables could significantly predict group classification (dynapenic vs. sarcopenic). All results are expressed as mean ± SD. The level of significance was set at *p* < 0.05.

## 3. Results

A total of 164 participants (female, *n* = 96, 59%) were included in this study. Comparisons among the non-diseased (female = 34, male = 34), dynapenic (female = 34, male = 25), and sarcopenic (female = 28, male = 9) groups revealed significant differences in age, weight, and height. Specifically, the sarcopenic group was older and had lower weight and height than the other groups. The HGS, and 5TSTS values were significantly better in the non-diseased group than in the other groups (*p* < 0.001); and 5TSTS differed significantly between dynapenic and sarcopenic groups. The SMI was significantly lower in the sarcopenic group than in the non-diseased or dynapenic groups (*p* < 0.001) (Table 1).

### 3.1. Comparison of Spatiotemporal GV Among the Non-Diseased, Dynapenic, and Sarcopenic Groups

Compared with the non-diseased group, the dynapenic and sarcopenic groups showed significantly longer stance phase time (*p* < 0.001), load response time (*p* < 0.001), and double stance time (*p* < 0.001) and significantly reduced stride length (*p* < 0.001), gait speed (*p* < 0.001), and cadence (*p* < 0.001). However, no significant differences in step width were observed among the three groups and none in any of the gait variables between the dynapenic and sarcopenic groups. In terms of variability, the stance phase time (*p* < 0.001), stride length (*p* < 0.001), gait velocity (*p* < 0.001), and cadence (*p* < 0.001) exhibited significantly greater variability in both dynapenic and sarcopenic groups than in the non-diseased group. In contrast, the CV for step width was significantly higher in the sarcopenic group than in the non-diseased group and in the sarcopenic group than in the dynapenic group (*p* < 0.002). After adjusting for sex and age as covariates, significant group differences in the spatiotemporal gait parameters and most variability measures remained; however, the significance for stride length CV disappeared after adjustment (Table 2).

### 3.2. Comparison of Physical Function and Muscle Thickness and Stiffness Among the Non-Diseased, Dynapenic, and Sarcopenic Groups

In terms of muscle thickness, the BF, TA, and GAmed were significantly thinner in both dynapenic and sarcopenic groups than in the non-diseased group (*p* < 0.001), whereas the RF and GAlat were significantly thinner in the sarcopenic group than in the non-diseased group (*p* < 0.001). Additionally, the RF, TA, and GAmed were significantly thinner in the sarcopenic group than in the dynapenic group (*p* < 0.001). Regarding contractile stiffness, the RF, BF, and GAlat showed lower values in both dynapenic and sarcopenic groups than in the non-diseased group (*p* < 0.001). GAmed was significantly lower in the sarcopenic group than in the non-diseased group; there was also a significant difference between dynapenic and sarcopenic groups (*p* < 0.001). For relaxed stiffness, the BF and GAmed showed significantly higher values in the sarcopenic group than in the non-diseased group, with significant differences between the dynapenic and sarcopenic groups. After adjusting for sex as a covariate, no significant effect of age and sex were found on the differences among the three groups (Table 3).

## 4. Discussion

This study compared non-diseased older adults with those classified as dynapenic and sarcopenic, focusing on differences in physical function, spatiotemporal gait parameters, GV, and lower limb muscle characteristics, including muscle thickness and contractile and relaxed stiffness. Both dynapenia and sarcopenia negatively affected HGS, 5TSTS, gait, and muscle function. In particular, sarcopenia was associated with a greater decline in the overall physical performance, impaired gait stability, and structural and mechanical alterations in specific muscles. Moreover, dynapenia and sarcopenia can be distinguished based on GV, particularly step width CV, as well as differences in the thickness of the RF, TA, GAmed, and GAlat muscles; contractile stiffness of the GAmed; and relaxed stiffness of both BF and GAmed. Although sex was included as a covariate, it did not significantly influence the observed group differences. This suggests that the differences in muscle characteristics among the three groups were independent of sex.

Walking ability, including gait speed, stride length, and cadence, is an effective and reliable indicator of physical function in older adults [37], and spatiotemporal gait variables have been suggested as useful tools for the early detection of sarcopenia [38]. In this study, older adults with dynapenia and sarcopenia showed increased stance, double stance, and load response times as well as decreased stride length, velocity, and cadence compared to the non-diseased group. In addition, GV increased across all parameters. These findings suggest that older adults with dynapenia and sarcopenia exhibited more cautious and inefficient gait patterns and that the increased variability in gait parameters reflects reduced gait stability and impaired motor control [39]. These results confirm that muscle weakness, rather than muscle mass, is a major risk factor for the decline in walking ability [40]. In addition, in this study, there was a difference in the step width CV between the dynapenic and sarcopenic groups. Step width is a parameter that determines the size of the base of support during walking, and its variability implies that it is more sensitive to lateral instability [41]. This suggests that those with sarcopenia may experience greater difficulty in maintaining lateral stability during walking than those with dynapenia and may be accompanied by a more pronounced gait instability.

The sarcopenic group exhibited significantly reduced RF, BF, TA, GAmed, and GAlat muscle thickness compared with the non-diseased group. These findings suggest that lower extremity muscle thickness serves as a morphological indicator and also as a composite marker, reflecting both functional capacity (particularly muscle strength) and structural attributes (muscle mass). Key muscles, including the RF, BF, TA, and GAmed, play essential roles in walking, standing, and maintaining balance [42]. A reduction in the thickness of these muscles is therefore indicative not of simple physiological aging, but of functional decline and pathological progression of sarcopenia, supporting findings of previous research [43,44]. Importantly, our findings suggest that decreased thickness of the posterior muscles, including the BF, TA, and Gamed, may serve as an early indicator of muscle function decline.

This suggests that muscle thickness reduction can be used as an early indicator of muscle function decline. In particular, atrophy of the RF, TA, GAmed, and GAlat muscles starts at the dynapenia stage and gradually worsens as sarcopenia progresses; this can be interpreted as a pathophysiological continuum reflecting progressive neuromuscular function decline. The authors suggest that this atrophy progression trajectory could provide important clues for effective intervention timing and target muscle groups to prevent or delay sarcopenia.

In the analysis of muscle stiffness, the sarcopenic group showed significant reductions in contractile stiffness of the RF, BF, GAmed, and GAlat muscles. As stiffness, measured using SWE, correlates positively with muscle contractility [45], decreased contractile stiffness reflects reduced muscle strength, which is closely associated with functional decline. Notably, while HGS did not differ significantly between the dynapenic and sarcopenic groups, the 5TSTS revealed significant differences. This suggests that lower limb strength loss plays a more critical role in functional impairment than upper limb strength loss and that contractile muscle stiffness may serve as a more sensitive early marker. Thus, a decrease in contractile stiffness measured using SWE is closely associated with functional decline. Furthermore, the distinction between dynapenia and sarcopenia appears to be more strongly related to reductions in lower-limb muscle strength than in upper-limb strength. These findings support previous evidence that muscle strength, rather than muscle mass, is directly linked to functional deterioration [46]. In addition to the decline in muscle quality, reductions in muscle mass are also closely associated with impaired physical function in older adults. Recent evidence indicates that lower skeletal muscle mass is significantly correlated with lower Short Physical Performance Battery scores, and poorer overall functional performance [47,48]. Therefore, both quantitative (muscle mass loss) and qualitative (reduction in muscle stiffness) changes in skeletal muscle appear to contribute to functional decline. In this study, we found reduced contractile stiffness of the RF, BF, GAm, and GAl in dynapenic and sarcopenic groups. These findings suggest that decreased contractile stiffness in the lower-limb muscles may serve as an early indicator of functional decline and dynapenia.

Additionally, in this study, the relaxed stiffness of the BF and GAmed differed between the dynapenic and sarcopenic groups. In general, an increase in collagen fibers increases muscle stiffness [17]. Skeletal muscle is composed of muscle fibers embedded within intramuscular connective tissue (IMCT), which consists of the epimysium, perimysium, and endomysium [49]. The passive mechanical properties of skeletal muscles are largely determined by the structure and composition of the connective tissue [50]. As aging progresses, IMCT thickness increases [51], leading to elevated passive muscle stiffness [52,53]. Furthermore, aging induces both physical and biological changes in the connective tissue, including increased stiffness of collagen [54]. As collagen fibers are inherently inelastic, their accumulation reduces the muscle’s ability to recover elastically after stretching, resulting in increased resting tension and muscle stiffness [55]. Based on this mechanism, normal muscles remain soft and compliant in a relaxed state, whereas aging muscles may show elevated passive stiffness due to collagen accumulation [54,56]. In this study, the decrease in contractile stiffness of the BF and GAmed muscles may serve as a valuable indicator of sarcopenia and dynapenia.

These findings suggest that evaluating the contractile stiffness of the GAmed and GAlat, along with the relaxed stiffness of the BF and GAmed, may provide useful supplementary markers for the early detection of muscle dysfunction. In particular, changes in GAmed stiffness may be especially informative for predicting functional impairment associated with dynapenia.

Despite these important findings, this study has several limitations. First, due to its cross-sectional design, causal relationships among muscle strength, muscle mass, and functional decline could not be clearly established. Second, the participants were community-dwelling older adults recruited from a single region; therefore, caution is required when generalizing these findings to broader populations. Third, although SMI, HGS, and the 5TSTS were assessed, other indicators of muscle quality, such as muscle fascia thickness, were not evaluated. Fourth, muscle thickness and stiffness were measured at a single time point, which prevented the examination of temporal trends or progression patterns. Fifth, important confounding factors—such as physical activity levels, nutritional status, and comorbidities including diabetes and hypertension—were not assessed. Previous studies have shown that diabetes and hypertension are associated with decreased muscle mass, muscle quality, and strength [57,58], and physical activity and nutritional status are known to influence muscle properties [59,60]. Given that these factors affect muscle mass, muscle stiffness, and functional performance, the absence of such data limits the ability to fully adjust for potential confounding and may affect the interpretation of the results. Future studies should incorporate these variables to provide a more comprehensive understanding of age-related muscle decline. Future research should employ large-scale longitudinal studies that incorporate key influencing factors—such as diverse age groups, varying levels of physical activity, and the coexistence of other diseases—to better elucidate the multifactorial nature of muscle deterioration and its causal pathways.

## 5. Conclusions

This study confirmed that there were significant differences in gait variability, muscle thickness, and stiffness among older adults classified as non-diseased, dynapenic, and sarcopenic. In particular, step width variability and the contractile and relaxed stiffness of the GAmed appeared to be promising factors for differentiating between dynapenia and sarcopenia. These findings indicate that evaluating both quantitative and mechanical properties of muscle, in addition to strength and mass, may enhance the understanding of age-related functional decline. Further longitudinal research is warranted to clarify the predictive value of these indicators and to support their clinical application in early screening, fall risk prevention, and the development of individualized rehabilitation approaches for older adults.

## Figures and Tables

**Table 1:** General characteristics and body composition of participants.

	Non-Diseased (n = 68)	Dynapenic (n = 59)	Sarcopenic (n = 37)	Between Group Differences	
				F	*p*
Sex (male/female)	28 (41.2)/40 (58.8)	28 (47.5)/31 (52.5)	17 (45.9)/20 (54.1)		
Age (years)	76.67 ± 4.39	82.18 ± 5.20	82.24 ± 5.15	25.680	<0.001 ^ab^
Weight (kg)	64.47 ± 8.26	60.46 ± 7.29	59.96 ± 7.47	5.454	0.005 ^ab^
Height (cm)	162.02 ± 8.37	158.12 ± 7.18	156.29 ± 8.03	8.071	<0.001 ^ab^
MMSE-K	27.35 ± 2.20	25.54 ± 2.29	25.14 ± 2.41	15.119	<0.001 ^ab^
Hand grip strength (kg)	28.99 ± 8.84	17.88 ± 6.18	15.94 ± 5.84	52.630	<0.00 ^ab^
5TSTS (s)	9.36 ± 1.55	17.89 ± 5.28	16.55 ± 3.31	96.193	<0.001 ^abc^
SMI (kg/m^2^)	7.26± 0.93	7.01 ± 0.80	5.54 ± 0.67	53.545	<0.00 ^bc^

K-MMSE: Korean version of the Mini-Mental State Examination, 5TSTS: five times sit-to-stand, SMI, skeletal muscle index. ^a^ *p* < 0.05 indicate a significant difference between non-diseased and dynapenic groups. ^b^ *p* < 0.05, significant difference between non-diseased and sarcopenic groups. ^c^ *p* < 0.05 indicate a significant difference between dynapenic and sarcopenic groups.

**Table 2:** Comparison of spatiotemporal gait variables and variability between non-diseased, dynapenic, and sarcopenic groups.

	Non-Diseased (n = 68)	Dynapenic (n = 59)	Sarcopenic (n = 37)	Between Groups			
				F	*p*	Adj *p* ^a^	Adj *p* ^b^
Stance phase (%)	65.90 ± 2.40 (65.62–66.48)	70.28 ± 4.67 (69.06–71.05)	69.83 ± 5.91 (67.86–71.80)	19.544	<0.001 ^ab^	0.002	0.005
Stance phase CV (%)	2.46 ±1.37 (2.13–2.80)	4.15 ± 2.74 (3.43–4.86)	3.84 ± 2.43 (2.82–5.02)	8.662	<0.001 ^ab^	<0.001	0.004
Load response time (%)	15.68 ± 2.37 (15.10–16.28)	19.70 ± 3.84 (18.70–20.70)	19.19 ± 5.32 (17.42–20.97)	20.981	<0.001 ^ab^	<0.001	0.008
Load response time CV (%)	9.55 ± 4.59 (8.44–10.66)	11.41 ± 5.96 (9.86–12.97)	10.87 ± 6.21 (8.80–12.95)	1.921	0.150	0.337	0.121
Double stance time (%)	31.34 ± 4.36 (30.28–32.39)	39.57 ± 7.50 (39.63–41.54)	38.74 ± 10.74 (35.16–42.33)	23.254	<0.001 ^ab^	0.001	0.037
Double stance time CV (%)	7.43 ± 6.77 (5.79–9.07)	8.16 ± 3.52 (7.24–9.08)	7.97 ± 3.87 (6.68–9.27)	0.335	0.716	0.156	0.113
Stride length (cm)	79.19 ± 23.73 (73.45–84.94)	55.79 ± 17.66 (51.19–60.40)	60.38 ± 18.22 (62.97–70.09)	22.693	<0.001 ^ab^	<0.001	0.001
Stride length CV (%)	5.05 ± 2.93 (4.34–5.76)	9.86 ± 6.66 (8.12–11.59)	10.05 ± 9.91 (6.75–13.35)	11.422	<0.001 ^ab^	0.061	0.029
Step width (cm)	10.95 ± 2.93 (10.23–11.66)	10.83 ± 3.09 (10.02–11.64)	11.66 ± 3.97 (10.33–12.98)	0.808	0.447	0.648	0.120
Step width CV (%)	15.82± 6.08 (14.35–17.29)	18.82± 11.37 (15.86–21.79)	22.97 ± 12.73 (18.73–27.22)	6.285	0.002 ^bc^	0.012	0.007
Velocity (km/hour)	2.86 ± 0.89 (2.64–3.08)	1.72 ± 0.60 (1.57–0.88)	1.87 ± 0.60 (1.67–2.07)	42.723	<0.001 ^ab^	<0.001	0.007
Velocity CV (%)	4.52 ± 2.42 (3.93–5.11)	10.07 ± 6.29 (8.42–11.71)	10.89 ± 12.58 (6.70–15.09)	13.278	<0.001 ^ab^	0.012	0.002
Cadence (step/min)	120.91 ± 15.00 (117.28–124.55)	104.48 ± 17.72 (99.86–109.10)	104.80 ± 22.42 (97.32–112.27)	16.580	<0.001 ^ab^	0.002	0.031
Cadence CV (%)	3.07 ± 1.79 (2.63–3.50)	5.92 ± 4.25 (4.81–7.03)	5.66 ± 4.91 (4.02–7.30)	11.457	<0.001 ^ab^	0.001	0.004

Mean ± Standard deviation (95% confidence intervals), CV: coefficient of variation. ^a^ *p* < 0.05 indicates a significant difference between non-diseased and dynapenic groups. ^b^ *p* < 0.05 indicates a significant difference between non-diseased and sarcopenic groups. ^c^ *p* < 0.05 indicates a significant difference between dynapenic and sarcopenic groups. Adj *p* ^a^: *p*-value adjusted for age using analysis of covariance. Adj *p* ^b^: *p*-value adjusted for sex using analysis of covariance.

**Table 3:** Comparison of muscle thickness and stiffness between non-diseased, dynapenic, and sarcopenic groups.

	Non-Diseased (n = 68)	Dynapenic (n = 59)	Sarcopenic (n = 37)	Between Groups			
				F	*p*	Adj *p* ^a^	Adj *p* ^b^
RF thickness (mm)	19.12 ± 3.54 (18.26–19.98)	18.14 ± 3.89 (17.13–19.16)	15.62 ± 3.45 (14.47–16.77)	11.078	<0.001 ^bc^	0.013	0.003
relaxed stiffness (kPa)	26.38 ± 7.72 (24.51–28.25))	27.67 ± 10.36 (24.97–30.37)	27.71 ± 9.47 (24.55–30.87)	0.406	0.667	0.347	0.086
contractile stiffness (kPa)	100.36 ± 31.14 (92.81–107.90)	81.68 ± 31.42 (73.49–89.87)	71.77 ± 29.18 (62.04–81.50)	11.777	<0.001 ^ab^	0.030	0.008
BF thickness (mm)	29.44 ± 5.96 (27.99–30.88)	23.55 ± 6.97 (21.74–25.37)	21.43 ± 8.82 (19.39–23.47)	23.252	<0.001 ^ab^	<0.001	<0.001
relaxed stiffness (kPa)	18.97 ± 4.30 (17.93–20.01)	20.94 ± 7.51 (19.00–22.92)	24.37 ± 8.14 (21.43–27.31)	7.686	<0.001 ^bc^	0.004	0.021
contractile stiffness (kPa)	37.13 ± 18.18 (32.73–41.53)	26.77 ± 13.54 (22.75–30.80)	26.45± 13.54 (21.94–30.97)	8.264	<0.001 ^ab^	0.015	0.009
TA thickness (mm)	23.32 ± 4.06 (22.34–24.31)	21.25 ± 4.36 (20.11–22.39)	19.28 ± 3.96 (17.96–20.60)	11.799	<0.001 ^ab^	0.032	<0.001
relaxed stiffness (kPa)	30.10 ± 8.85 (27.95–32.24)	29.27 ± 6.83 (27.49–31.05)	31.11 ± 9.54 (27.93–34.30)	0.557	0.574	0.353	0.689
contractile stiffness (kPa)	153.38 ± 17.33 (149.18–157.57)	149.05 ± 20.93 (143.59–154.50)	152.92 ± 16.80 (147.32–158.52)	0.955	0.387	0.827	0.088
GAmed thickness (mm)	16.33 ± 2.13 (15.81–16.84)	15.46 ± 2.76 (14.74–16.18)	13.44 ± 2.23 (12.70–14.19)	17.390	<0.001 ^bc^	0.005	0.022
relaxed stiffness (kPa)	17.02 ± 4.01 (15.94–18.11)	17.34 ± 4.01 (16.30–18.39)	20.38 ± 7.31 (17.94–22.81)	5.687	0.004 ^bc^	0.287	0.002
contractile stiffness (kPa)	90.30 ± 28.27 (83.46–97.15)	81.39 ± 26.54 (74.47–88.31)	68.76 ± 30.04 (58.74–78.70)	7.097	<0.001 ^bc^	0.031	0.010
GAlat thickness (mm)	13.75 ± 2.84 (13.06–14.44)	13.22 ± 3.14 (12.40–14.04)	11.15 ± 2.09 (10.45–11.85)	10.583	<0.001 ^bc^	0.006	<0.001
relaxed stiffness (kPa)	19.08 ± 6.58 (17.48–20.67)	18.26 ± 5.72 (16.77–19.76)	20.65 ± 6.20 (18.58–22.72)	1.688	0.188	0.135	0.333
contractile stiffness (kPa)	99.13 ± 31.04 (91.61–106.64)	73.14 ± 31.30 (64.99–81.30)	79.10 ± 29.22 (69.36–88.84)	12.243	<0.001 ^ab^	0.027	0.008

Mean ± Standard deviation (95% confidence intervals), RF, rectus femoris; BF: Biceps femoris; TA: Tibialis anterior; GAmed: Gastrocnemius medialis; GAlat; Gastrocnemius lateralis. ^a^ *p* < 0.05 indicate a significant difference between non-diseased and dynapenic groups. ^b^ *p* < 0.05 indicate a significant difference between non-diseased and sarcopenic groups. ^c^ *p* < 0.05 indicate a significant difference between dynapenic and sarcopenic groups. Adj *p* ^a^: *p*-value adjusted for age using analysis of covariance. Adj *p* ^b^: *p*-value adjusted for sex using analysis of covariance.

## Data Availability

The data presented in this study are available on request from the corresponding author.
